# The influence of incorporation of hydroxyapatite/collagen nanocomposite into glass ionomer cement on surface roughness, microhardness, and fluoride-ion release potential

**DOI:** 10.1186/s12903-025-07080-1

**Published:** 2025-11-01

**Authors:** Mostafa A. Abdelshafi, Ahmed Elsebaai, Kareem Hamdi

**Affiliations:** 1 Dental Biomaterials Department, Faculty of Dentistry, Mansoura University, Mansoura, Egypt; 2 Faculty of Oral and Dental Medicine, Alsalam University, Tanta, Egypt; 3 Pediatric Dentistry Department, Faculty of Oral and Dental medicine, Delta University for Science and Technology, Gamasa, Egypt; 4 Conservative Dentistry Department, Faculty of Oral and Dental Medicine, Zagazig University, Zagazig, 35516 Egypt

**Keywords:** Glass ionomer cement, Hydroxyapatite, Collagen, Nanocomposite, Surface roughness, Microhardness, Fluoride-ion release

## Abstract

**Objective:**

To investigate the effect of incorporation of 5 and 10 weight% of hydroxyapatite /collagen (HA/Col) nanocomposite with different ratios (50:50) and (70:30) to conventional glass ionomer cement (C-GIC) on surface roughness, microhardness, and fluoride-ion release.

**Materials and methods:**

Seventy-five specimens were prepared using split Teflon mold (5 mm in diameter and 2 mm in thickness) and were randomly assigned to 5 different groups. Group I: control group of C-GIC, Group II: GIC modified with 5% HA/Col (50:50), Group III: GIC modified with 5% HA/Col (70:30), Group IV: GIC modified with 10% HA/Col (50:50), Group V: GIC modified with 10% HA/Col (70:30). The specimens were kept in deionized (DI) water and incubated at 37 °C.The DI water in each container was changed on the 1st, 7th, 14th, and 28th days. Fluoride ion release was assessed using the SPADNS colorimetric technique. Afterwards, a surface profilometer and a Vickers hardness tester were used to assess surface roughness and microhardness. Moreover, micromorphological analysis was assessed using SEM-EDX mapping.

**Results:**

Regarding fluoride-ion release, mixed linear model revealed significant increase of fluoride-ion release in the 4 experimental groups compared to control one (*p* < 0.0001). Regarding surface roughness, and microhardness, one-way ANOVA test revealed a significant increase of surface roughness of experimental group of 10% HA/Col (50:50) compared to rest of groups, and significant increase of surface microhardness of all experimental groups compared to control one.

**Conclusion:**

Incorporation of HA/Col nanocomposite into C-GIC cement showed promising improvements in surface hardness and fluoride ion release.

**Supplementary Information:**

The online version contains supplementary material available at 10.1186/s12903-025-07080-1.

## Background

Recently, a diverse array of materials that release fluoride has been introduced in restorative dentistry, including fluoride-incorporated composites, polyacid modified-composite resins, as well as glass ionomer cement [1]. Recurrent caries remains one of the most common causes of failure in dental restorations [2]. In this context, fluoride-releasing restorative materials have demonstrated potential in reducing the risk of recurrent caries, as supported by various studies in the dental literature [3]. Glass ionomer cement (GIC) is a commonly used restorative material that contains calcium or strontium aluminum fluorosilicate glass powder in conjunction with soluble co-polymers. In the presence of water, these components undergo an acid-base reaction [4]. Clinicians’ preference for GICs is attributed to their sustained release of fluoride ions. It has been reported that conventional GIC (C-GIC) releases fluoride at levels five times greater than compomers and 21 times greater than a fluoride-incorporated composite resins [5]. Furthermore, GIC can bond chemically to calcified dental structure [6]. Conversely, several shortcomings have been reported about GIC as a permanent restoration including insufficient fracture toughness, compressive strength, hardness, and wear resistance [6, 7]. The abovementioned drawbacks of GIC have driven the scientific community to solve this dilemma.

Various attempts were conducted to improve mechanical properties of GIC. In this regard an increase in the powder-to-liquid ratio and reduction in glass particle size showed improved mechanical properties compared to C-GIC [8]. Furthermore, resin-modified glass ionomer cements (RMGICs) are light-cured materials that contain the same components as conventional GICs, with the addition of the hydrophilic resin monomer 2-hydroxyethyl methacrylate (2-HEMA) and a camphorquinone photo-initiator system [9]. Superior mechanical properties of RMGICs compared to conventional GICs have been stated in extensive dental research [10–13]. In contrast, another research evidence stated that the addition of resins to the GICs did not enhance the surface microhardness of these materials [14]. Moreover, several published studies have shown promising mechanical properties of GICs with the incorporation of various fillers into the cement powder, including hydroxyapatite (HA), titanium dioxide, cellulose, aluminosilicate fibers, carbon, zinc, zirconia, and silver [15–20].

HA is a synthetic inorganic structure similar to biologic apatite of the bone and teeth which has osteoconductive and bioactive properties making its use more favorable in restorative dentistry [21]. Previously published studies have shown that the inclusion of HA in GICs resulted in enhanced mechanical properties, along with its capability for remineralisation and antibacterial effects [21]. Additionally, both collagen and HA have been widely utilized in tissue engineering due to their high bioactivity [22]. In earlier dental research, the source of collagen was the fibrous portion of bovine flexor tendon to resemble human type I collagen presents in human periodontal tissue [23]. The composite structure of these two natural materials was assumed to be superior compared to a monolithic one. The ductile nature of collagen enhances the fracture toughness of HA while reducing its stiffness, whereas the inclusion of HA in the collagen matrix boosts the composite’s mechanical stability [22].

A thorough review of the published literature revealed that several studies have investigated the effect of HA and nano-hydroxyapatite (NHA) particles on the mechanical properties of GIC, meanwhile, only one study has evaluated the mechanical properties of GIC enhanced with collagen integration [24]. Consequently, the current study was designed to fill such a gap. The research hypothesis of the current study was that significant differences would exist between GIC in the control group and GIC modified with different concentrations and ratios of hydroxyapatite/collagen (HA/Col) nanocomposite regarding surface roughness, microhardness, and fluoride-ion release.

## Materials and methods

### Study design

This study was designed and conducted at the Department of Restorative Dentistry (Delta University for Science and Technology, Egypt) in 2024. The local ethical committee of Delta University for Science and Technology reviewed and approved this in vitro study with the protocol number (DU:024100555) on April 5th 2024.

### Materials

Chemicals used in preparation of HA/Col nanocomposite and used glass ionomer cement in the study are described in details in Table 1.

**Table 1:** Materials used in the study

Materials	Chemical form/Composition	Batch No	Manufacturer
Calcium Nitrate Tetrahydrate	Ca (NO_3_)_2_. 4H_2_O	13477-34-4	Acros Organics, USA
Diammonium Hydrogen Phosphate	(NH_4_)_2_HPO_4_	7783-28-0	Fisher Bioreagents, USA
Collagen from Bovine Achilles Tendon	Type I Collagen	C9879	Sigma-Aldrich Chemie, Steinheim, Germany.
Sodium Hydroxide	NaOH	91,830	
Acetic Acid	CH_3_COOH	23.3131905	CHMI-LAB NV, Belgium
Medifil Glass Ionomer Filling Cement	Powder: Calcium fluoroaluminosilicate glass liquid: polyacrylic acid	2603	Promedica, Germany

### Methods

#### Preparation of self-assembled HA/Col nanocomposites

HA/Col nanocomposite was prepared following the preparation protocol conducted in a previously published study by Abdelshafi, MA. et al. [25]. The initial amounts of all reagents were scaled in consistent with the final HA/Col ratio of 50/50, and 70/30.

#### Characterization of synthetized HA/Col nanocomposite

HA/Col nanocomposite chemical functional groups were previously assessed in a laboratory study conducted by Abdelshafi, MA. et al. [25] using Attenuated Total Reflection Fourier Transform Infrared (ATR-FTIR) spectroscopy (Thermo Fisher Scientific, Nicolet iS10, USA). Additionally, the crystalline phases of hydroxyapatite were identified through X-ray diffraction (XRD) analysis and micro-morphological comparative analysis of synthesized HA/Col nanocomposite with different ratios were inspected by transmission electron microscope (TEM) [25].

### Sample size calculation

G* Power statistical software (ver. 3.0.10, Franz Faul, Universität Kiel, Germany) was used to calculate the sample size based on a reference study [26]. A statistical power of 95%, an α error of 5%, and f = 0.25 effect size. The number of samples required to be included in the study was 75 which were randomly assigned to 5 different groups (*n* = 15). The details of the groups for mechanical testing are shown in Table 2.

**Table 2:** Testing groups with their corresponding sample size

**Groups**	**Weight percentage % of added (HA-Col) nanocomposite**	**No of samples**
control	0	15
HA/Col (70/30) nanocomposites-GIC	5	15
	10	15
HA/Col (50/50) nanocomposites-GIC	5	15
	10	15

### Formulation of glass ionomer powder with HA/Col nanocomposite

HA/Col nanocomposites were weighed and incorporated into the commercial glass ionomer cement (Medifil Glass Ionomer Filling Cement, Promedica, Germany) powder at concentrations of 5%, and 10% by weight for the experimental groups. The mixtures were manually ground for 10 min using an agate mortar and pestle and thoroughly blended with the glass ionomer powder container. The nanocomposite was incorporated by replacement, whereby 5 wt% and 10 wt% of the GIC powder were substituted with HA/Col nanocomposite to produce homogeneously blended modified powders while maintaining the manufacturer’s recommended powder-to-liquid ratio. To ensure further uniform and homogeneous distribution of the prepared powder throughout the GIC powder, the mixture was placed into empty clean amalgam capsule and vibrated for about 20 s in an amalgamator (de Gotzen, softly 8). The powder-to-liquid ratio was determined following the manufacturer’s instructions (one scoop of powder was mixed with one drop of liquid). Then, the cement was left covered with moist gauze for up to 24 h to ensure complete setting. Afterwards, ATR-FTIR analysis (Thermo Fisher Scientific, Nicolet iS10, USA) was conducted on the GIC-HA/Col nanocomposite powder in the spectral range of 400–4000 cm⁻¹, with a resolution of 4 cm⁻¹, to assess the chemical bonds present in the samples including characteristic peak at 567, 606, 1023, and 1630 cm-^1^ spectra to ensure homogeneity of the mix. Additionally, the set glass ionomer cements from both the control and experimental groups were analyzed using ATR-FTIR as previously mentioned at the same spectral range.

### Samples preparations

A split Teflon mold measuring (5 mm diameter × 2 mm height) was utilized for the preparation of the samples. The mold was initially placed on a glass plate and covered with a Mylar sheet. The materials were mixed and handled following the manufacturers’ guidelines, then inserted into the molds with a slight excess. A Mylar sheet was placed over the mold, which was then topped with a second glass plate. Gentle pressure was applied to eliminate most of the excess cement extruded during molding, the samples were removed from the molds and immediately finished and polished using a complete sequence of aluminum oxide abrasive discs (TOR VM Ltd., Moscow, Russia). The discs were mounted on a latch-type mandrel attached to a low-speed handpiece, operating at 20,000 rpm under continuous water irrigation. The procedure was conducted in accordance with the manufacturer’s instructions, applying gentle pressure in a circular motion for 20 s per disc, with a new disc used for each specimen. The procedure was performed by a single operator to ensure standardization. Finally, samples were left covered with moist gauze for up to 24 h to ensure complete setting. Following that, they were then kept in deionized water at 37 ± 1 °C with 100% humidity until testing. To ensure operator blinding while performing tests, the samples were coded so that the operator couldn’t identify the experimental groups.

### Fluoride-ion release

Prepared disc-shaped specimens were suspended in 10 mL of deionized (DI) water in plastic containers and incubated at 37 ± 1 °C. The DI water in each container was changed on the 1 st, 7th, 14th, and 28th days. The SPADNS colorimetric approach (SM 4500 F standard method for testing of water and wastewater, 24th edition, 2023) was used to measure the fluoride contents in deionised water. The basic idea of this method is the interaction of fluoride ions with a zirconium-dye lake complex. When fluoride and the dye lake mix, the dye partially dissociates, forming a transparent complex anion (ZrF₆²⁻). The resulting colour gradually lightens as the fluoride concentration rises.

Preparing SPADNS reagent requires dissolving 958 mg (0.958 g) of SPADNS (Sulfanilic acid azochromotrop, Sigma-Aldrich, USA) in DI water and then diluting the solution to a final volume of 500 mL with DI water. Preparing zirconyl-acid reagent necessitates dissolving 133 mg (0.133 g) of zirconyl chloride octahydrate, ZrOCl₂·8 H₂O (Sigma-Aldrich, USA), in approximately 25 mL of DI water. After adding 350 mL of concentrated HCl, 500 mL of DI water is added to dilute the solution. Equal parts of the SPADNS solution and the zirconyl-acid reagent are combined to create the acid zirconyl-SPADNS reagent.

A set of six standard calibration solutions with fluoride concentrations of 0.0, 0.2, 0.4, 0.6, 0.8, and 1.0 ppm was prepared from a certified fluoride reference material (Merck KGaA, Darmstadt, Germany). The absorbance of the standards was recorded at 570 nm using a UV-visible double-beam spectrophotometer (LAMDA 365, PerkinElmer, USA). The samples, diluted at a 1:25 ratio, were then measured relative to the prepared standards.

### Surface roughness

After 28 days of storage in distilled water, surface roughness was measured utilizing a surface profilometer (Surftest 211, Mitutoyo, Tokyo, Japan). Calibration was performed using the reference specimen supplied by the manufacturer before each measurement session. Each specimen’s roughness was assessed at five distinct locations spaced at least 0.5 mm apart and ≥ 1.0 mm from the disc edge to avoid edge effects. A cut-off value of 0.8 mm was used for surface roughness, with the stylus covering a distance of 3.0 mm during each measurement. The tracing diamond tip has a radius of 5 μm, and the measuring force and speed were set to be 4 mN and 0.5 mm/s, respectively, under controlled laboratory conditions. The average roughness value (Ra, in µm) for each disc was determined by calculating the mean of the Ra values, which represent the average of surface peaks and valleys, measured at five different locations [27]. 

### Surface microhardness

Surface microhardness was measured utilizing Vickers Micro-hardness Tester (Wilson^®^ Tukon 1102/1202 Series; Buehler) equipped with a Vickers diamond indenter together with a 20X objective lens. The device was calibrated against the certified reference block supplied by the manufacturer before testing. Each sample was tested with a 50 g load applied by the diamond indenter, at a dwell time of 10 s. Three indentations were conducted on the surface of each specimen, with a minimum distance of 0.5 mm between them, and the mean value of these points was considered the final measurement. The lengths of the indentation diagonals were measured using the built-in calibrated microscope. The Vickers hardness number (VHN) was computed by dividing the applied load by the area of the indentation, as determined by the following equation:

$$\mathrm{VHN}=1.8544\times\mathrm P/\mathrm d^2$$

where P represents the applied load in kgf, and d denotes the diagonal length in mm [28]. 

### Scanning electron microscope and energy dispersive x-ray mapping analysis (SEM-EDX)

After completion of fluoride-ion release, surface roughness, and microhardness test, one specimen from each group was selected to be representative for micromorphological analysis using SEM with tungsten filament as electron source (TESCAN-VEGA-COMPACT, GmbH, Germany) at high voltage of 30 kV and magnification of 2000 X. Specimen preparation prior to micromorphological analysis was done via gold-coating using a sputter coater, with a gas pressure of approximately 50 mTorr and a current of around 40 MA. The length of coating process was about 180 s. Moreover, the size and distribution of nanocomposite particles within the set cement were investigated using a TESCAN VEGA COMPACT scanning electron microscope operated at 30 kV. Finally, to ensure the homogeneous distribution of nanoparticles within the modified GIC containing HA/Col nanocomposite, SEM coupled with an Energy-Dispersive X-ray (EDX) detector (TESCAN VEGA COMPACT, GmbH, Germany) was performed on one sample from each of the following groups: C-GIC, 10% HA/Col (50:50)-GIC, and 10% HA/Col (70:30)-GIC, for elemental mapping analysis.

### Statistical analysis and data interpretation

Data analysis was performed by SPSS software, version 26 (SPSS Inc., PASW statistics for windows version 26. Chicago: SPSS Inc.). Quantitative data were described using mean ± standard deviation (SD) for normally distributed data after testing normality using Shapiro Wilk test. Significance of the obtained results was judged at the (0.05) level.

- One Way ANOVA test was used to compare more than 2 independent groups with Post Hoc Tukey test to detect pair-wise comparison.
- mixed linear model test was used to study the combined effect of 2 independent factors on dependent continuous outcome with estimation of R^2^.

The raw data and quantitative data analysis are provided in Supplementary Files 1 and 2, respectively.

## Results

### Characterization of HA/Col nanocomposite mixed with glass ionomer cement powder

**Fig. 1:**
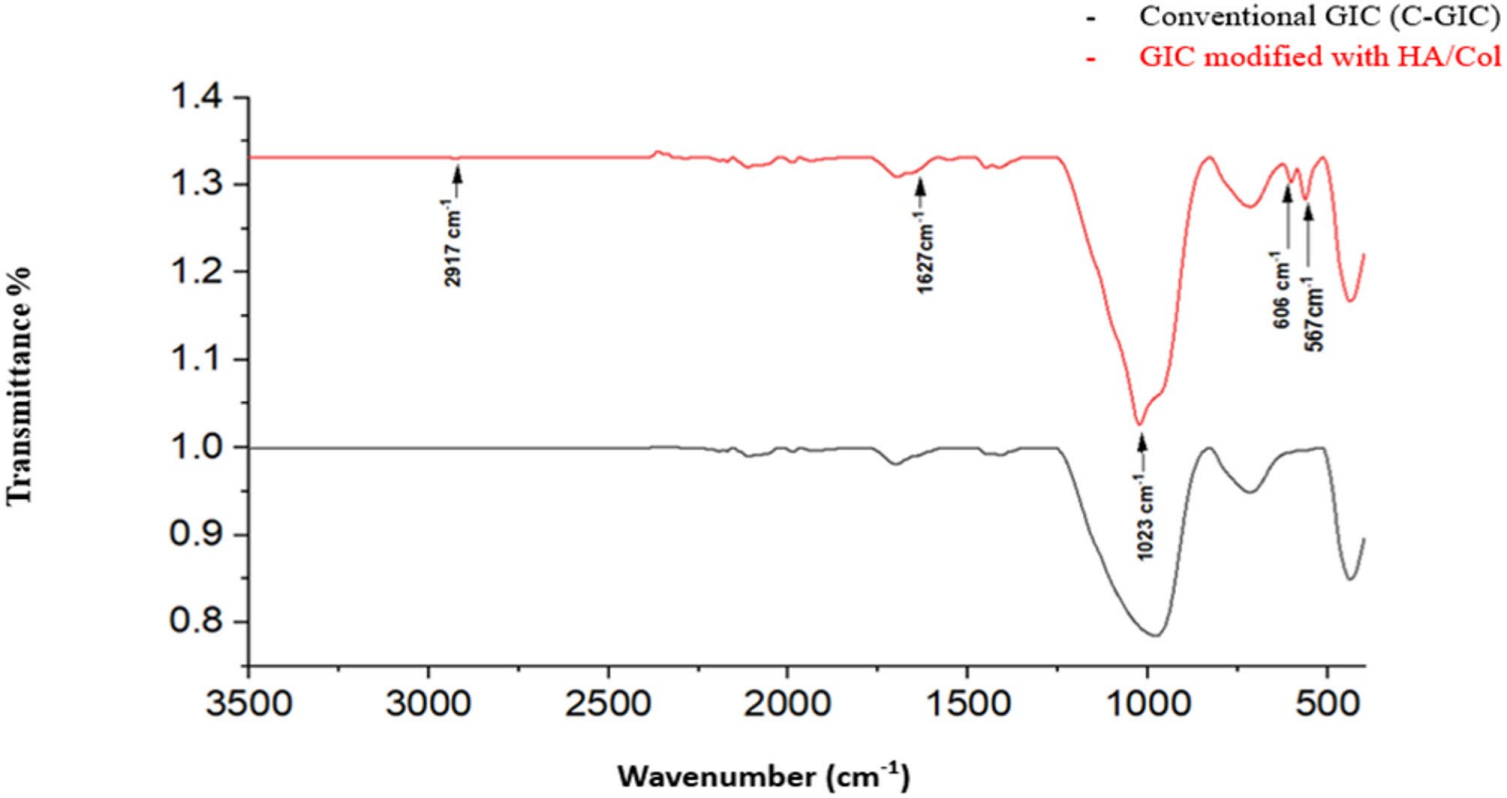
ATR-FTIR of conventional glass ionomer cement powder (C-GIC) (Black), and GIC powder modified with HA/Col nanocomposite (Red)

**Fig. 2:**
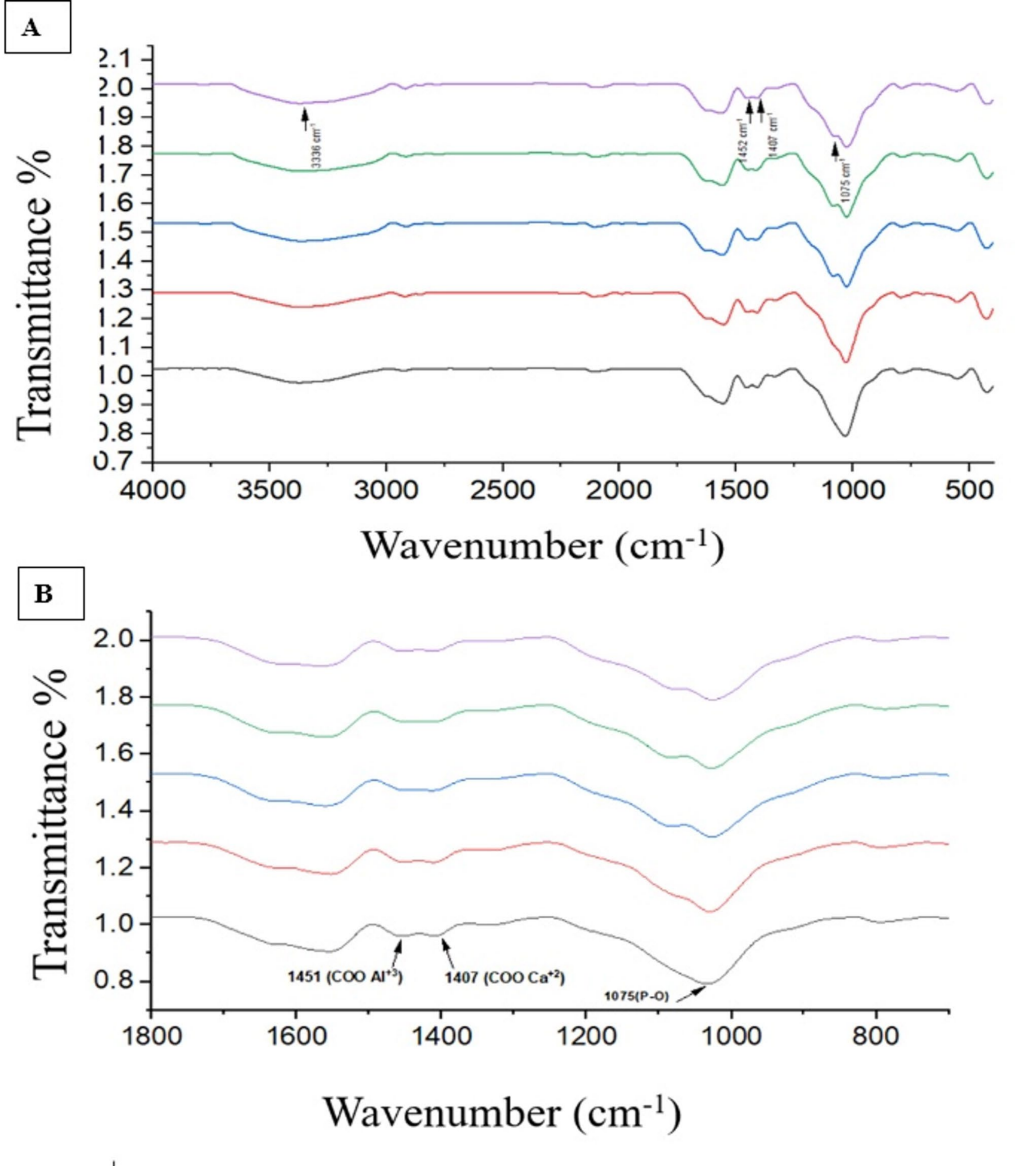
**A: **FTIR of set GIC of different groups. **B: **FTIR fingerprint spectral range from 800-1800 of set cement showed carboxylate salts were recognized at 1407, 1451 cm^-1^ respectively. (Note: Black: Control, Red: GIC-5%NHA/Col 50:50. Blue: GIC-10%NHA/Col 50:50. Green: GIC-5% NHA/Col 70:30. Violet: GIC-10% NHA/Col 70:30)

ATR-FTIR of HA/Col nanocomposite mixed with glass ionomer cement powder verified the presence of characteristic peaks indicating the existence of HA and collagen. A peak at 567 cm-^1^ spectra corresponding to *V*_*2*_ O-P-O bending mode, besides a characteristic band corresponding to *V*_*4*_ O-P-O bending mode at 606 cm-^1^ were detected. Additionally, a peak at 1023 cm − ^1^ corresponding to the vibrational mode of *V*_*3*_ asymmetric P-O stretching was also detected. Characteristic peaks of collagen were observed, including the C = O stretching at 1630 cm⁻¹ for amide I. These findings indicate the effective embedding of HA/Col nanocomposite within the conventional matrix. (Fig. 1) Finally, FTIR analysis for set glass ionomer cement in different groups was performed and hydroxyl groups (OH) from the water content of GIC liquid was recognized at 3336 cm^−1^. (Fig. 2A) Setting reaction showed progressive reduction in COOH groups to COO- groups which is corresponding to formation of metal salts. This was recognized in FTIR fingerprint spectral range from 800 to 1800 of set cement, accordingly, carboxylate salts were recognized at 1407, 1451 cm^−1^ respectively. (Fig. 2.B)

### Fluoride-ion release

Regarding fluoride release, mixed linear model revealed that all groups showed significant reduction of fluoride release after 28 days. (Table 3) A significant interaction between group and time was observed (*p* < 0.0001), indicating that fluoride release potential differed across materials. The control group exhibited a typical burst release with decreasing values over time (from 5.09 ppm on Day 1 to 3.11 ppm at 28 days). In contrast, all experimental HA/Col-containing groups demonstrated significantly higher fluoride release at each time point compared to the control (*p* < 0.0001). The 5% HA/Col (70:30) group recorded the highest initial release (12.62 ppm), while the 10% HA/Col (50:50) group showed the most sustained release between Day 7 and 14. (Table 4) These findings suggest that both the HA/Col ratios and concentration influence the fluoride release kinetics, with potential implications for optimizing long-term cariostatic effects.

**Table 3:** Mixed linear model for predictors of fluoride release

Source	Type III Sum of Squares	df	Mean Square	F	Sig.	Partial Eta Squared (effect size)
Corrected Model	2166.020^a^	19	114.001	3500.411	0.0001*	0.996
Intercept	13540.263	1	13540.263	415754.820	0.0001*	0.999
groups	453.696	4	113.424	3482.691	0.0001*	0.980
Time of assessment	1494.828	3	498.276	15299.599	0.0001*	0.994
groups * Time of assessment	217.497	12	18.125	556.521	0.0001*	0.960
Error	9.119	280	0.033			
Total	15715.403	300				
Corrected Total	2175.139	299				

a. R Squared = 0.996 (Adjusted R Squared = 0.996)

**Table 4:** Multiple comparisons of fluoride release between different groups at differentperiods

Groups * time assessment					
Dependent Variable: Fluoride					
groups	Time assessment	Mean	Std. Error	95% Confidence Interval	
				Lower Bound	Upper Bound
Control	28 Days	3.111	0.047	3.020	3.203
	14 Days	4.203	0.047	4.111	4.294
	7 Days	4.859	0.047	4.768	4.951
	1 Day	5.093	0.047	5.002	5.185
5% (70:30 HA: Col)	28 Days	4.564	0.047	4.472	4.656
	14 Days	6.452	0.047	6.360	6.544
	7 Days	7.551	0.047	7.460	7.643
	1 Day	12.618	0.047	12.526	12.710
10% (70:30 HA: Col)	28 Days	4.081	0.047	3.989	4.172
	14 Days	6.210	0.047	6.118	6.302
	7 Days	7.675	0.047	7.584	7.767
	1 Day	11.098	0.047	11.006	11.190
5% (50:50 HA: Col)	28 Days	3.571	0.047	3.479	3.662
	14 Days	6.422	0.047	6.330	6.514
	7 Days	7.380	0.047	7.288	7.472
	1 Day	10.553	0.047	10.462	10.645
10% (50:50 HA: Col)	28 Days	3.621	0.047	3.530	3.713
	14 Days	7.113	0.047	7.022	7.205
	7 Days	7.471	0.047	7.379	7.562
	1 Day	10.717	0.047	10.625	10.808

#### Effect size for fluoride-ion release

Effect sizes (partial eta squared) indicated very large effects for group (η² = 0.980), time (η² = 0.994), and their interaction (η² = 0.960), suggesting that both the material composition and time significantly impacted fluoride release behavior. (Table 3)

### Surface roughness and microhardness

A one-way analysis of variance (ANOVA) was conducted to examine the effect of the experimental groups on surface roughness and microhardness and significant differences were observed for both variables (*p* < 0.05). (Table 5) Then multiple comparisons between different groups have been conducted in relation to surface roughness (Table 6), and microhardness (Table 7) independently.

**Table 5:** Analysis of variance (ANOVA) results for microhardness and surface roughness across treatment groups

		Sum of Squares	df	Mean Square	F	Sig.
microhardness	Between Groups	2656.191	4	664.048	5.447	0.001
	Within Groups	8534.239	70	121.918		
	Total	11190.431	74			
roughness	Between Groups	0.723	4	0.181	8.884	0.000
	Within Groups	1.423	70	0.020		
	Total	2.146	74

**Table 6:** Comparison of roughness (Ra) between different studied groups

		*N*	Mean	Std. Deviation	Test of significance
Roughness	Control	15	.518^a^	0.19	F = 8.884 *p* = 0.001*
	5% HA/Col (70:30)	15	.429^b^	0.105	
	10% HA/Col (70:30)	15	.511^c^	0.106	
	5% HA/Col (50:50)	15	.473^d^	0.113	
	10% HA/Col (50:50)	15	.715^abcd^	0.169	

F: One Way ANOVA test, similar letters in same column denote significant different between studied groups by post Hoc Tukey test

**Table 7:** Comparison of microhardness between different studied groups

		*N*	Mean	Std. Deviation	Test of significance
Microhardness	Control	15	42.59^abcd^	4.67	F = 5.447 *P* = 0.001*
	5% HA/Col (70:30)	15	54.84^a^	10.88	
	10% HA/Col (70:30)	15	58.91^b^	16.89	
	5% HA/Col (50:50)	15	58.30^c^	8.802	
	10% HA/Col (50:50)	15	55.91^d^	10.32	

#### Surface roughness

Regarding surface roughness, one-way ANOVA test revealed that there was no significant difference between control group and the following experimental groups 5% HA/Col (70:30), 10% HA/Col (70:30), and 5% HA/Col (50:50). While, there was significant difference between the experimental group of 10% HA/Col (50:50) and the rest of groups (*P* < 0.05), as the highest surface roughness was detected in this group. (Table 6)

##### Effect size for One-Way ANOVA (Roughness)

One-way ANOVA showed a statistically significant difference between groups (F = 8.884, *p* = 0.001, η² = 0.34), indicating a large effect size. Tukey’s HSD post hoc test (adjusted for multiple comparisons) revealed that only the 10% HA/Col (50:50) group differed significantly from the rest, showing the highest surface roughness.

**Eta Squared (η²) was calculated using the following formula**:

η2 = Sum of Squres (SS) between/SS total

From the current data:

Surface Roughness.

- F = 8.884, *p* = 0.001
- No SS values given, but we can estimate η² from F, df_between = 4, and df_within = 70 (15 samples × 5 groups = 75 total → df_total = 74, df_within = 70)

Using the formula:

$$\mathrm\eta^2=\frac{\mathrm F\cdot{\mathrm{df}}_{\mathrm{between}}}{\mathrm F\cdot{\mathrm{df}}_{\mathrm{between}}+{\mathrm{df}}_{\mathrm{within}}}=\frac{8.884\cdot4}{8.884\cdot4+70}=\frac{35.536}{105.536}\approx0.337$$

η**²** ≈ 0.34 → large effect

#### Surface microhardness

Regarding surface microhardness of glass ionomer and glass ionomer samples one-way ANOVA test demonstrated that there was a significant increase of surface microhardness of experimental groups compared to control one. However, there was no significant difference between the four experimental groups. The highest surface microhardness was recorded in glass ionomer cement modified with 10% HA/Col (70:30). (Table 7)

##### Effect size for One-Way ANOVA (Microhardness)

A significant increase in the microhardness was observed in all experimental groups compared to the control (F = 5.447, *p* = 0.001, η²=0.24) indicating a large effect size. Tukey’s HSD post hoc test confirmed that while all HA/Col groups outperformed the control, differences between the experimental groups themselves were not statistically significant.

**Microhardness**

- F = 5.447, *p* = 0.001
- Same group size, so:
  - df_between = 4, df_within = 70
    $$\mathrm\eta^2=\frac{5.447\cdot4}{5.447\cdot4+70}=\frac{21.788}{91.788}\approx0.237$$

**η² ≈** 0.24 → large effect

### SEM/EDX mapping results

**Fig. 3:**
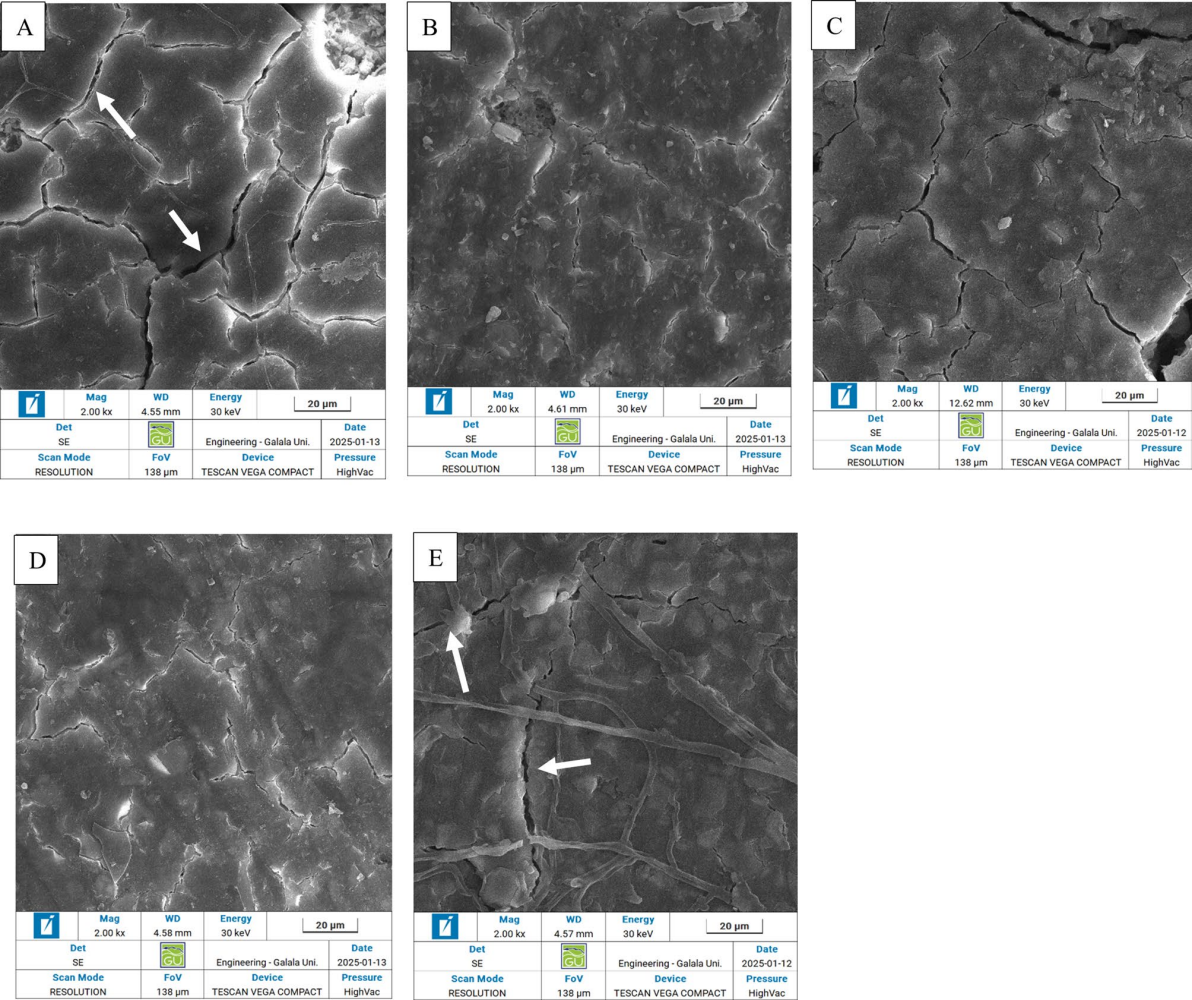
**A**: Control group showed marked generated cracks (white arrows). **B**: Experimental group of 10% GIC-HA/Col (70:30) showed prominent crystal deposition partially sealing the generated cracks. **C**: Experimental group of 5% GIC-HA/Col (70:30) showed crystal precipitation and shrouded minor cracks (white arrows). **D**: Experimental group of 10% GIC-HA/Col (50:50) showed marked crystal deposition partially sealing the generated cracks.** E**: Experimental group of 5% GIC-HA/Col (50:50) showed crystal precipitation and shrouded exposed minor cracks (white arrows)

**Fig. 4:**
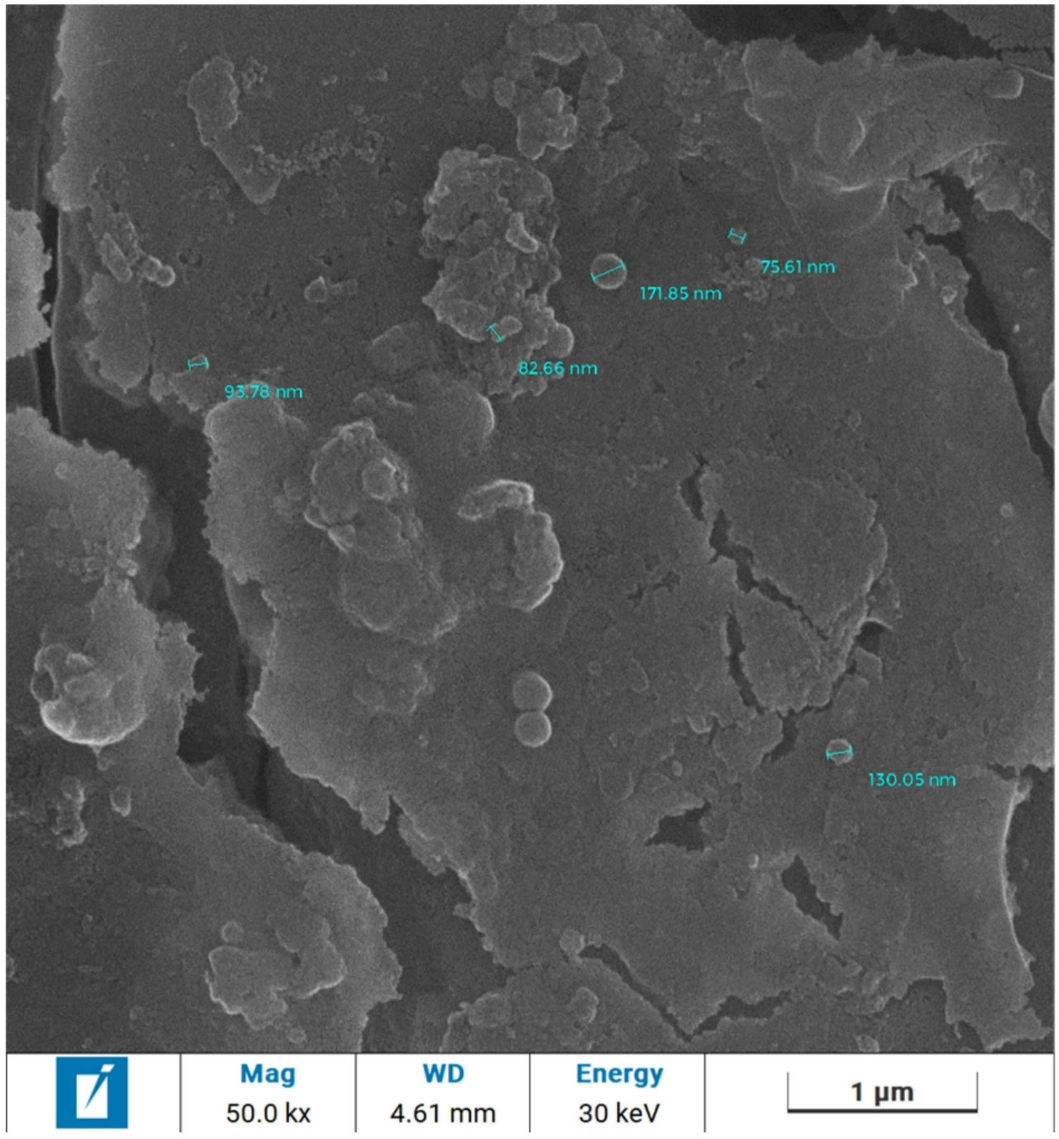
Scanning electron microscopy image of synthesized nano-sized hydroxyapatite/ collage nanocomposite in set cement showing particle size in the range of 70—130 nm, forming agglomerates

The surface of all GIC samples in different group was depicted in Fig. 3. All groups exhibited cracks on the surface of the samples. The control group showed prominent crack lines in the surface of sample (Fig. 3A), while the experimental groups showed crystals precipitates on the surface of GIC samples. Crystals precipitation was prominent in experimental group of 10% GIC-HA/Col (70:30) and 10% GIC- HA/Col (50:50) (Fig. 3B, D) compared to 5% GIC-HA/Col (70:30) and 5% GIC-HA/Col (50:50) (Fig. 3C, E). Scanning electron microscopy image of synthesized nano-sized hydroxyapatite/collage in set cement showing particle size in the range of 70–130 nm, forming agglomerates. (Fig. 4)

**Fig. 5:**
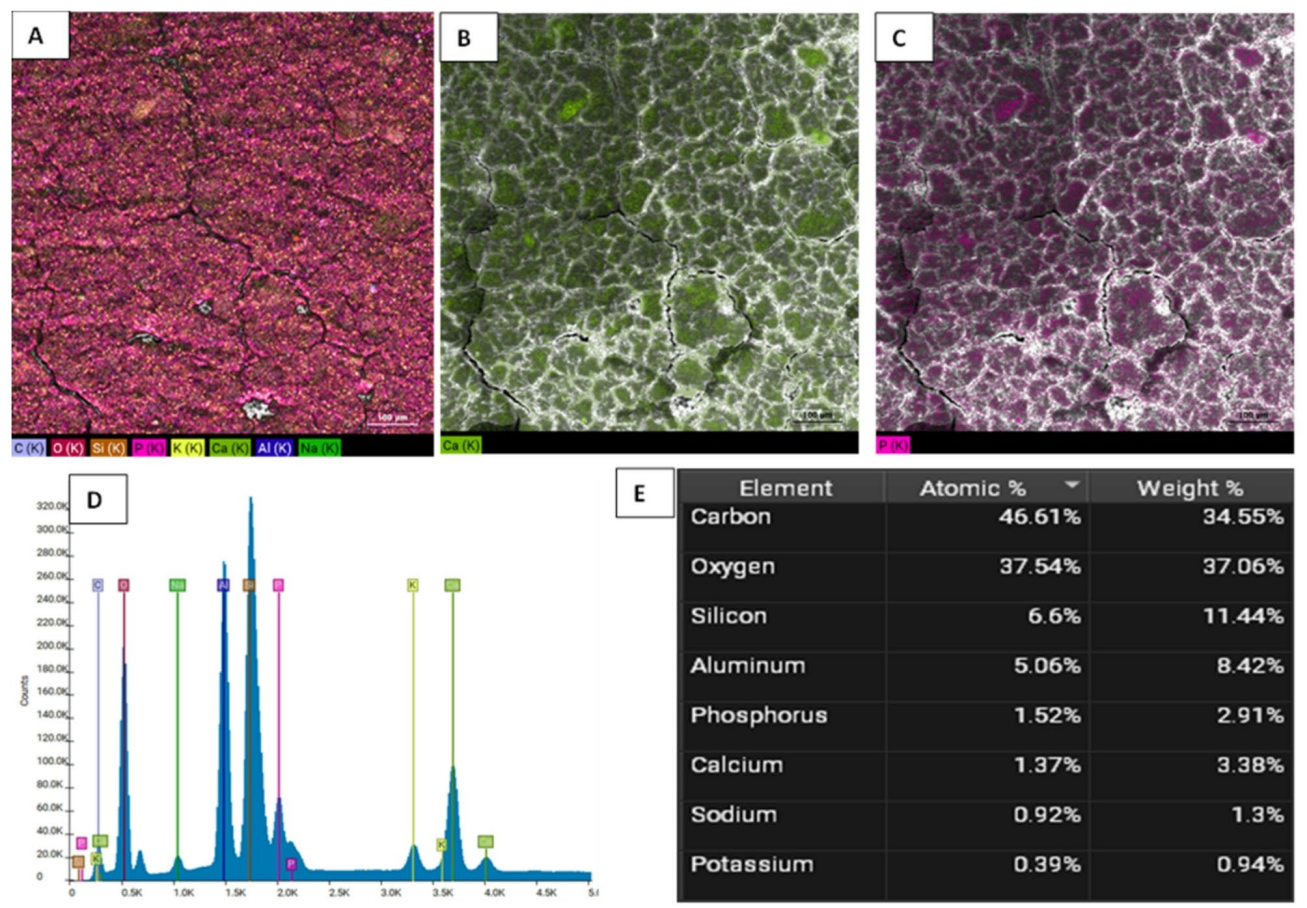
**A**: Elemental mapping of GIC-10% HA/Col (70:30). **B**: EDX mapping of Calcium. **C**: EDX mapping of Phosphorus. **D**: Elemental spectrum. **E**: Quantitative analysis (Table Weight Fraction)

**Fig. 6:**
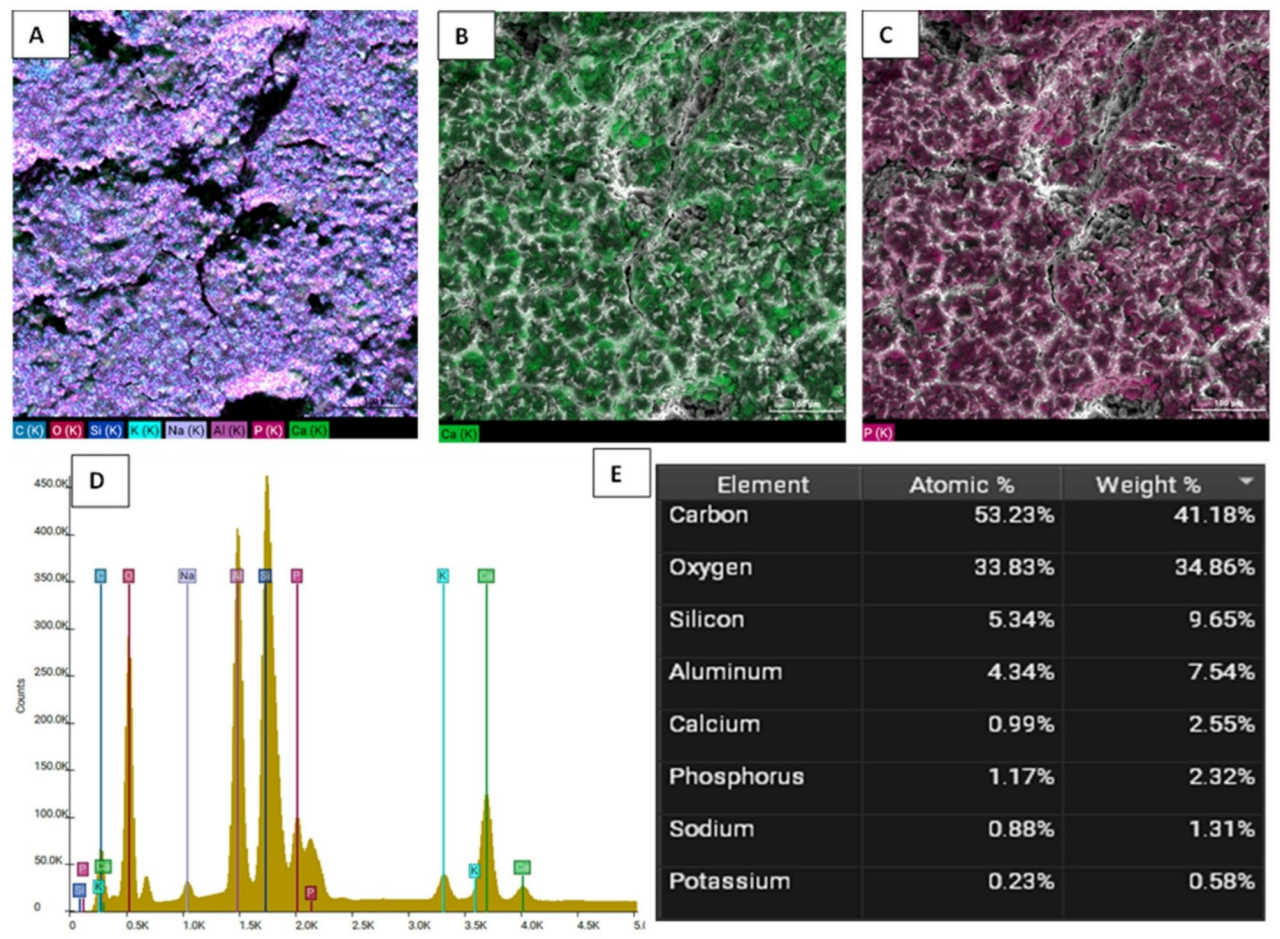
**A**: Elemental mapping of GIC-10% HA/Col (50:50). **B**: EDX mapping of Calcium. **C**: EDX mapping of Phosphorus. **D**: Elemental spectrum.** E**: Quantitative analysis (Table Weight Fraction)

**Fig. 7:**
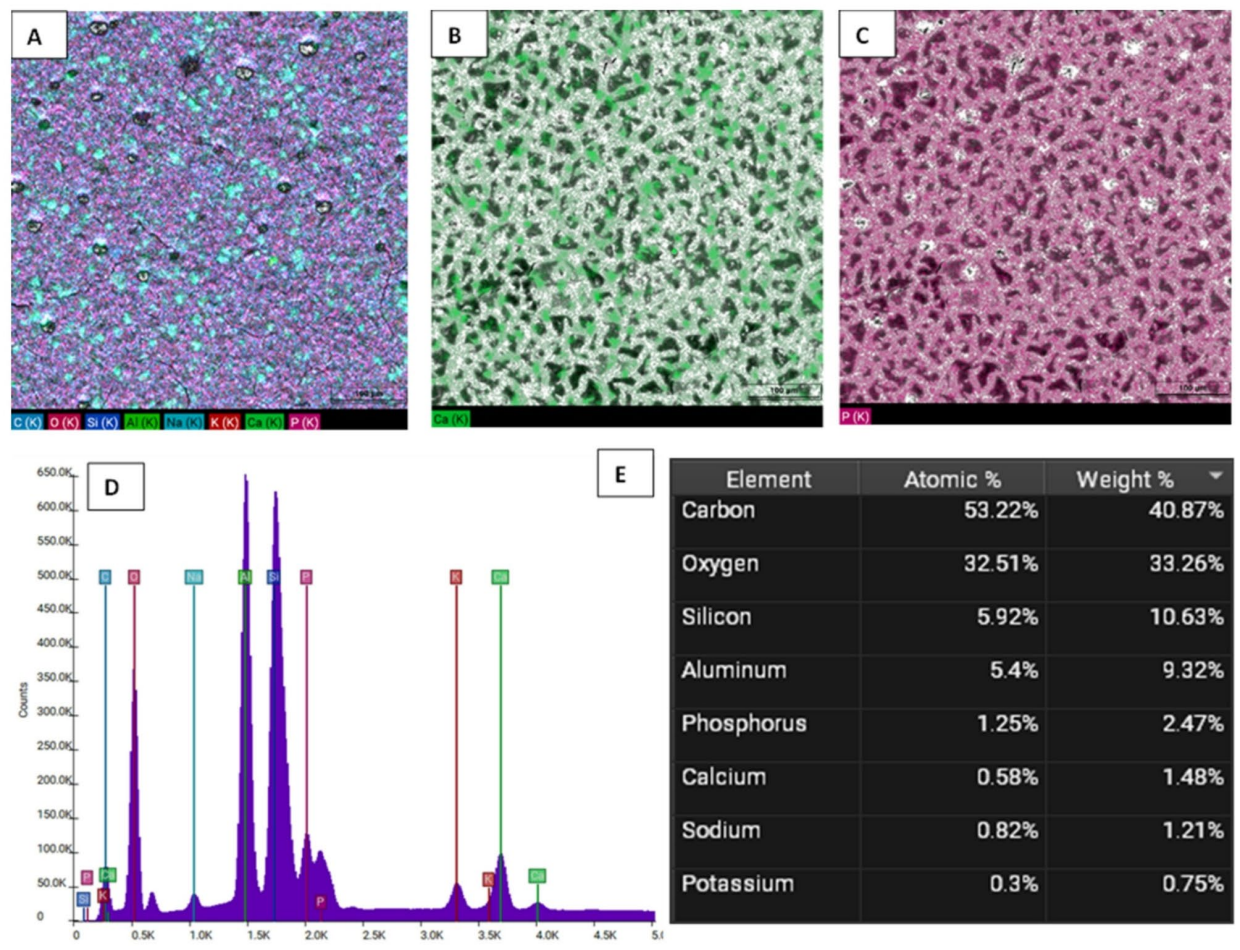
**A**: Elemental mapping of C-GIC (Control). **B**: EDX mapping of Calcium. **C**: EDX mapping of Phosphorus. **D**: Elemental spectrum.** E**: Quantitative analysis (Table Weight Fraction)

Figures 5 and 6, and 7 illustrate the EDX elemental mapping, spectra, and quantitative elemental analysis of the three representative samples: 10% GIC-HA/Col (70:30), 10% GIC-HA/Col (50:50), and the control, respectively. These analyses provide insights into the spatial distribution and relative abundance of key elements, particularly calcium (Ca) and phosphorus (P), which are crucial indicators of mineral phase distribution within the composite matrix.

In terms of calcium distribution, the 10% GIC-HA/Col (70:30) sample (Fig. 5B) exhibits a more uniform and homogeneous dispersion of Ca throughout the matrix. This contrasts with the 10% GIC-HA/Col (50:50) sample (Fig. 6B), where Ca appears less uniformly distributed, and the signal intensity is comparatively reduced. In the control sample (Fig. 7B), calcium is highly localized and sporadically distributed, supporting the observed quantitative reduction in Ca content and indicating poor mineral integration.

Regarding phosphate distribution, P appears as clustered regions in all samples. However, the 10% GIC-HA/Col (70:30) sample (Fig. 5C) shows the highest density and most extensive phosphate-rich zones. This suggests a stronger and more coherent mineral phase in this composition, potentially enhancing bioactivity and mechanical integration.

Quantitative EDX analysis further supports these observations:

- The 10% GIC-HA/Col (70:30) sample demonstrates a well-balanced Ca/P distribution with a near-ideal atomic ratio of ~ 1.16 (Fig. 5E), consistent with the expected stoichiometry of hydroxyapatite and indicating efficient mineral incorporation.
- In the 10% GIC-HA/Col (50:50) sample, although Ca and P co-localize spatially, the overall Ca signal is reduced, resulting in a slightly lower Ca/P ratio of ~ 1.10 (Fig. 6E), suggesting suboptimal mineral distribution compared to the 70:30 formulation.
- The control sample exhibits a high phosphate signal but limited and discontinuous calcium presence, reflected in a markedly low Ca/P ratio of ~ 0.60 (Fig. 7E).

## Discussion

The research hypothesis of the current study was totally accepted as there were significant differences between C-GIC and GIC modified with different ratios and concentrations of HA/Col nanocomposite regarding surface roughness, microhardness, and fluoride-ion release. Numerous efforts have been reported in dental research to address the aforementioned limitations of C-GICs, including the incorporation of various fillers—such as glass, metal, and other non-reactive particles—into the GIC matrix [29]. Additionally, several attempts have been explored to enhance the bioactivity of the material by incorporation of HA [Ca _10_ (PO _4_) _6_ (OH) _2_]. The remarkable biological properties of HA are ascribed to its similarity to the structure and composition of natural apatite found in the human hard tissue [29, 30]. Several published studies have evaluated the impact of addition of HA to GIC powder on mechanical properties of GIC and reported promising outcomes [15, 31–33]. At the level of nano-scale, it was reported that incorporation of nano-HA particles (NHA) into GIC powder showed remarkable improvement in mechanical properties. This was ascribed to remarkable increase in surface area, in addition to powerful penetration of the nano-crystals into enamel and dentine surfaces compared to micro crystals [34]. The majority of published studies evaluated the mechanical properties of modified GIC with NHA at the level of compressive and flexural strength. Furthermore, it was found that only a single study that has evaluated the mechanical properties of GIC enhanced with collagen integration [22]. Therefore, the present study was designed to evaluate the influence of varying concentrations and ratios of HA/Col nanocomposite on the surface roughness, microhardness, and fluoride-ion release of GIC, with the aim of developing a novel composite material suitable for a range of restorative applications. Different ratios of HA/Col nanocomposite (50:50 and 70:30) were selected as fillers to optimize the functional properties of the material. The 50/50 ratio provides a balanced mix of bioactivity and mechanical support, while the 70/30 ratio, with higher hydroxyapatite content, aims at improving material’s, hardness, wear resistance, and long-term durability in dental restorations. In contrast, the 30/70 ratio of HA/Col was excluded, as too much collagen may neutralize the pH of the matrix and decrease the ion exchange throughout the reaction, compromising the mechanical properties [23]. 

Regarding surface microhardness, the incorporation of HA/Col nanocomposite with different ratios and different concentrations into GIC showed a significant increase in surface microhardness compared to the control group. This can be justified by the high crystallinity of the glass structure of glass ionomer after addition of HA. Calcium ions of HA are displaced, and further precipitated in the acid base reaction. Furthermore, Hydrogen ions H^+^ from polymeric acids attacks the hydroxyapatite particles in the polyacid matrix and enhance cross-linking forming rigid, acid-resistant intermediate layer which might increase the surface hardness of the material [35, 36]. This outcome is in the same vein with Zhu, K. et al. [37] who stated that the enhanced surface hardness of GIC could attributed to homogenous distribution of NHA between flour aluminosilicate particles. Accordingly, this supplies polyacrylic acid with further bonding sites to form salt bridging, and crosslinking. The incorporation of collagen fibers in GIC was inspired from a previous laboratory study conducted by Chang H.-J. et al. [23]. They stated that, nano-collagen fibrous material could enhance the mechanical properties of GIC by the way of reinforcing the interconnection between silica fillers. Additionally, in orthopedic literature it was claimed that the overall stiffness and strength of the bone could be ascribed to the interfacial bonding between mineral phase and organic collagen matrix [38]. Notably, HA particles have a great affinity to collagen fibers via adsorption adhesion. Accordingly, the incorporation of HA/Col nanocomposite in the current study could be justified.

Moreover, the surface of all GIC samples in different group is shown in Fig. 3. All groups exhibited cracks on the surface of the samples which might be attributed to acidic attack of flouro-alumino-silicate matrix with polymeric acids or stresses generated during molding and sample preparation. Marked crystals precipitation in experimental group of 10% GIC-HA/Col (70:30) and 10% GIC- HA/Col (50:50) (Fig. 3B, D) justifies the superior surface hardness of these groups.

Surface roughness is an essential property to be evaluated in the restorative material as it affects the surface topography and has a crucial influence on esthetic outcome. Furthermore, it can be considered as one of antecedents to bacterial adherence that might contribute to further development of secondary caries. The findings of the current study showed that there was significant difference between the experimental group of 10% GIC-HA/Col (50:50) nanocomposite and the rest of groups, as the highest surface roughness was detected in this group. Such an observed increase in surface roughness of this group could be attributed to loss of the surface layer secondary to storage in distilled water, leading to exposure of further glass particles. These findings are in a total agreement with previously published laboratory study by Moheet, I. et al. [39]. They reported that the surface roughness of GIC modified with 10% by weight of nano-HA-Si increased after 28 days compared to the first day. Additionally, such findings are consistent with Chang H.-J. et al. [23] as they reported that the addition of too much collagen in glass ionomer cement significantly compromised the bonding between the matrix and the filler with increasing the susceptibility of glass particles exposure and this could justify the exclusion of the experimental group of HA/Col (30:70) from the study. In addition to that, heavy loading of GIC with 10% HA/Col (50:50) nanocomposite, with higher percentage of dense mineralized collagen bundles, might render them to be exposed as well after surface dissolution. Moreover, another factor that might justify the increased surface roughness is using conventional GIC in powder liquid form rather than using encapsulated form. However following manufacturer’s instruction in powder and liquid mixing, manual mixing may incorporates air voids which may alter the polymer conversion and introduces voids in the surface [40].

Fluoride-ion release is a distinct property of GIC that give it a biologic privilege over other tooth-colored restoration. Fluoride ions release plays crucial role in reduction the incidence of development of recurrent carious lesions. Additionally, released fluoride ions from restorative materials precipitated as CaF^2^ in the oral environment, functioning as a reservoir that releases additional fluoride ions when the oral pH decreases [41]. GIC has a slow releasing potential of fluoride ions from sample into liquid medium which has been clearly explained by two different mechanisms in published literature [41]. The first mechanism is a short-term process that involves the rapid dissolution of fluoride ions from the outer surface into the solution. Meanwhile, the second mechanism is a long-term process, driven by the continuous diffusion of ions throughout the bulk material. Additionally, fluoride ion releasing potential of GIC varies from storage medium to other. It was reported that, GIC showed higher fluoride releasing capacity when stored in de-ionized water compared to artificial saliva [41, 42]. Consequently, this seems a clear justification of using de-ionized water as a storage medium in the current study. The findings of the current study showed significant difference between the different groups after 28 days, as the highest amount of fluoride-ions release was detected in both groups of GIC- modified with 5%, and 10% HA/Col (70:30) nanocomposite compared to other groups. Such an increase of fluoride-ions release in these groups might be ascribed to presence of NHA particles and collagen which have high surface area and contribute in acid-base reaction, thus enhancing fluoride-ions release [43]. The release of fluoride could be catalyzed by collagen, resulting in a rapid and sustained diffusion of fluoride through the GIC matrix. The distinct cationic nature of collagen enables the formation of multilayer structures or electrostatic complexes with other natural or synthetic polymers, rendering it highly suitable for fluoride release in drug delivery applications. When polyacrylic acid adsorbs onto collagen, it forms reinforced complexes, together with NHA particles, that attach to the surface of the GIC particles. The formation of these complexes appears to enhance the release of fluoride ions from the inorganic matrix. The findings of the current study in this regard are in a total agreement with a previously published laboratory studies [26, 43]. It worth mentioning that, in all groups, the concentration of released fluoride ions was much lower after 28 days than it was on the first day. This has been well justified and clarified in published literature, as GIC releases the greatest amount of fluoride within the first 24 h and this phenomenon is called “Early burst phenomenon” [41].

Although 28 days is considered a relatively short period for evaluating fluoride release potential—a limitation that was addressed in the study’s limitations section—microhardness and surface roughness were assessed at this time point due to the presumed negative correlation between fluoride release and the material’s mechanical properties [44]. 

The elemental mapping and quantitative analysis of Ca and P revealed clear compositional differences among the tested samples, with important implications for the biomineralization potential and structural integrity of the developed composites. Notably, the 10% GIC-HA/Col (70:30) formulation exhibited a more homogeneous distribution of calcium and phosphate, along with a Ca/P ratio (~ 1.16) closely resembling that of natural hydroxyapatite (~ 1.67) [45], albeit slightly lower. In contrast, the 50:50 composition displayed a less intense and more heterogeneous calcium distribution, reflected in a slightly reduced Ca/P ratio (~ 1.10). This suggests that increasing collagen content may interfere with mineral phase homogeneity, possibly due to altered ion exchange dynamics or reduced availability of HA-binding sites.

These findings underscore the importance of optimizing the HA-to-collagen ratio in hybrid composites to achieve favorable elemental distribution and mineral phase stability. The superior performance of the 70:30 composition highlights its potential for dental applications where biomimetic mineralization is critical. Future work may explore the long-term stability of this composition under physiological conditions and its interaction with cellular environments.

For proper characterization of the experimental material, ATR-FTIR has been used and its usage is well-justified. FTIR absorbs light at different energy levels, which is then converted into vibrational frequencies representing the molecular fingerprint of the sample. Each molecule or chemical structure has a distinct fingerprint within a specific spectral range, making FTIR analysis a dependable tool for chemical characterization. ATR-FTIR has a history of being used for direct, and real-time analysis of the chemical composition of GIC powder and detection of the changes in bonding nature through the setting time. Additionally, XRD is a widely recommended method for material characterization, praised in dental literature for its ability to provide a clear identification of the mineral phase’s chemical composition, along with valuable information on the material’s crystallinity [46]. 

### Limitations

This study has certain limitations that should be acknowledged. First, it was conducted under in-vitro conditions, which cannot fully replicate the complex oral environment, including pH fluctuations, salivary enzymes, thermal changes, and masticatory forces. Therefore, direct generalization of the results to clinical performance should be made with caution. Second, the evaluation period was limited to short-term storage and testing intervals. Longer observation periods are needed to determine the stability of the mechanical and ion-release properties over time. Finally, while the incorporation of HA/Col nanocomposite improved specific material properties, the potential effects on other clinically relevant parameters, such as wear resistance, esthetics, and long-term bond durability, were not assessed. Another major limitation of the current study that must be emphasized is the absence of evaluation of the cytotoxic effect of the synthesized xenogeneic material which is a crucial step prior to translate such promising findings to clinical research. Future studies, particularly long-term in-vivo and clinical trials, are recommended to validate and extend these findings.

## Conclusion

Incorporation of 5–10% HA/Col nanocomposite with different concentrations either (70:30) or (50:50) into C-GIC showed promising results at level of surface microhardness which may extend the lifespan of restoration clinically. However, a higher collagen content may lead to an increase in surface roughness. Moreover, such a modification of GIC showed potential fluoride-ion release which will enhance the remineralization power and positive alternation of microbial and acidic environment during management of deep carious lesions. Future laboratory-based investigations incorporating long-term follow-up are warranted to validate and extend these findings.

F: One Way ANOVA test, similar letters in same column denote significant different between studied groups by post Hoc Tukey test.

## Supplementary Information

Supplementary Material 1.

Supplementary Material 2.

## Data Availability

The data set has been uploaded as a supplementary file.

## Abbreviations

GIC: Glass Ionomer Cement
RMGIC: Resin modified glass ionomer cement
HEMA: Hydroxyethyl methacrylate
C-GIC: Conventional glass ionomer cement
HA: Hydroxyapatite
NHA: Nano-hydroxyapatite
HA/Col: Hydroxyapatite/collagen
XRD: X-ray diffraction
EDX: Energy dispersive x-ray
SEM: Scanning electron microscope
ATR-FTIR: Attenuated total reflection fourier transform infrared
TEM: Transmission electron microscope
Ca/P: Calcium/phosphate ratio

## References

[1] Hicks J, Garcia-Godoy F, Donly K, Flaitz CJJCDA. Fluoride-releasing restorative materials and secondary caries. J Calif Dent Assoc. 2003;31(3):229–43. 12693822

[2] Demarco FF, Cenci MS, Montagner AF, de Lima VP, Correa MB, Moraes RR, Opdam NJM. Longevity of composite restorations is definitely not only about materials. Dent Mater. 2023;39(1):1–12. 36494241 10.1016/j.dental.2022.11.009

[3] Francois P, Fouquet V, Attal JP, Dursun E. Commercially available Fluoride-Releasing restorative materials: A review and a proposal for classification. Mater (Basel Switzerland). 2020;13(10):2313. 10.3390/ma13102313. 10.3390/ma13102313 PMC7287768 32443424

[4] Nicholson JW, Sidhu SK, Czarnecka B. Enhancing the mechanical properties of Glass-Ionomer dental cements: A review. Mater (Basel Switzerland). 2020;13(11):2510. 10.3390/ma13112510. 10.3390/ma13112510 PMC7321445 32486416

[5] Aboush YE, Torabzadeh H. Fluoride release from tooth-colored restorative materials: a 12-month report. J Can Dent Assoc. 1998;64(8):561–4. 9785685

[6] Sidhu SK, Nicholson JW. A review of Glass-Ionomer cements for clinical dentistry. J Funct Biomater 2016, 7(3). 10.3390/jfb7030016 PMC5040989 27367737

[7] Peutzfeldt A, García-Godoy F, Asmussen E. Surface hardness and wear of glass ionomers and compomers. Am J Dent. 1997;10(1):15–7. 9545914

[8] Šalinović I, Stunja M, Schauperl Z, Verzak Ž, Ivanišević Malčić A. Brzović Rajić V: mechanical properties of high viscosity glass ionomer and glass hybrid restorative materials. Acta Stomatol Croat. 2019;53(2):125–31. 31341320 10.15644/asc53/2/4 PMC6604565

[9] Mitra SB. Adhesion to dentin and physical properties of a light-cured glass-ionomer liner/base. J Dent Res. 1991;70(1):72–4. 1991864 10.1177/00220345910700011201

[10] Santos M, Leon L, Siddique I, Butler S. Retrospective clinical evaluation of RMGIC/GIC class V restorations. Dent J (Basel). 2023;11(9):211. 10.3390/dj11090211. 10.3390/dj11090225 PMC10529511 37754345

[11] Pereira LC, Nunes MC, Dibb RG, Powers JM, Roulet JF, Navarro MF. Mechanical properties and bond strength of glass-ionomer cements. J Adhes Dent. 2002;4(1):73–80. 12071632

[12] Momoi Y, Hirosaki K, Kohno A, McCabe JF. Flexural properties of resin-modified hybrid glass-ionomers in comparison with conventional acid-base glass-ionomers. Dent Mater J. 1995;14(2):109–19. 8940550 10.4012/dmj.14.109

[13] Xie D, Yang Y, Zhao J, Park JG, Zhang JT. A novel comonomer-free light-cured glass-ionomer cement for reduced cytotoxicity and enhanced mechanical strength. Dent Mater. 2007;23(8):994–1003. 17049978 10.1016/j.dental.2006.09.001

[14] Ellakuria J, Triana R, Mínguez N, Soler I, Ibaseta G, Maza J, García-Godoy F. Effect of one-year water storage on the surface microhardness of resin-modified versus conventional glass-ionomer cements. Dent Mater. 2003;19(4):286–90. 12686292 10.1016/s0109-5641(02)00042-8

[15] Gu YW, Yap AU, Cheang P, Khor KA. Effects of incorporation of HA/ZrO(2) into glass ionomer cement (GIC). Biomater. 2005;26(7):713–20. 10.1016/j.biomaterials.2004.03.019 15350775

[16] Boyd D, Towler MR. The processing, mechanical properties and bioactivity of zinc based glass ionomer cements. J Mater Sci Mater Med. 2005;16(9):843–50. 16167113 10.1007/s10856-005-3578-1

[17] Menezes-Silva R, de Oliveira BMB, Fernandes PHM, Shimohara LY, Pereira FV, Borges AFS, Buzalaf MAR, Pascotto RC, Sidhu SK, de Lima Navarro MF. Effects of the reinforced cellulose nanocrystals on glass-ionomer cements. Dent Mater. 2019;35(4):564–73. 30711272 10.1016/j.dental.2019.01.006

[18] Gjorgievska E, Nicholson JW, Gabrić D, Guclu ZA, Miletić I, Coleman NJ. Assessment of the impact of the addition of nanoparticles on the properties of Glass-Ionomer cements. Mater (Basel Switzerland). 2020;13(2):276. 10.3390/ma13020276. 10.3390/ma13020276 PMC7014475 31936253

[19] Poosti M, Ramazanzadeh B, Zebarjad M, Javadzadeh P, Naderinasab M, Shakeri MTJE. Shear bond strength and antibacterial effects of orthodontic composite containing TiO2 nanoparticles. 2013, 35(5):676–9. 10.1093/ejo/cjs073 23264617

[20] Bilić-Prcić M, Rajić VB, Ivanišević A, Pilipović A, Gurgan S, Miletić I. Mechanical Properties of Glass Ionomer Cements after Incorporation of Marine Derived Porous Cuttlefish Bone Hydroxyapatite. Materials (Basel, Switzerland). 2020;13(16):3614. 10.3390/ma13163614. 10.3390/ma13163542 PMC7475982 32796624

[21] Wan Jusoh WN, Matori KA, Mohd Zaid MH, Zainuddin N, Ahmad Khiri MZ, Abdul Rahman NA, Abdul Jalil R, Kul E. Incorporation of hydroxyapatite into glass ionomer cement (GIC) formulated based on Alumino-Silicate-Fluoride glass ceramics from waste materials. Mater (Basel Switzerland). 2021;14(4):806. 10.3390/ma14040806. 10.3390/ma14040954 PMC7923024 33670465

[22] Wang J, Liu CJJBE. Biomimetic collagen/hydroxyapatite composite scaffolds: fabrication and characterizations. 2014, 11(4):600–9.

[23] Chang HJ, Wu CM, Chang YC, Fanchiang JC, Shieh DB, Wong TY. Collagen enhances compatibility and strength of glass ionomers. J Dent Res. 2009;88(5):449–54. 19493889 10.1177/0022034509337478

[24] Chang H-J, Wu C-M, Chang Y-C, Fanchiang J-C, Shieh D-B. Wong T-YJJodr: collagen enhances compatibility and strength of glass ionomers. 2009, 88(5):449–54. 10.1177/0022034509337478 19493889

[25] Abdelshafi MA, Fathy SM, Elkhooly TA, Reicha FM, Osman MF. Bond strength of demineralized dentin after synthesized collagen/hydroxyapatite nanocomposite application. J Mech Behav Biomed Mater. 2021;121:104590. 34077907 10.1016/j.jmbbm.2021.104590

[26] Alatawi RAS, Elsayed NH, Mohamed WS. Influence of hydroxyapatite nanoparticles on the properties of glass ionomer cement. J Mater Res Technol. 2019;8(1):344–9.

[27] Bala O, Arisu HD, Yikilgan I, Arslan S, Gullu A. Evaluation of surface roughness and hardness of different glass ionomer cements. Eur J Dent. 2012;6(1):79–86. 22229011 PMC3252813

[28] Moheet IA, Luddin N, Ab Rahman I, Kannan TP, Abd Ghani NRNJCI. Evaluation of mechanical properties and bond strength of nano-hydroxyapatite-silica added glass ionomer cement. Ceram Int. 2018;44(8):9899–906.

[29] Moheet IA, Luddin N, Rahman IA, Kannan TP, Nik Abd Ghani NR, Masudi SM. Modifications of glass ionomer cement powder by addition of recently fabricated Nano-Fillers and their effect on the properties: A review. Eur J Dent. 2019;13(3):470–7. 31280484 10.1055/s-0039-1693524 PMC6890502

[30] Farooq I, Moheet IA, AlShwaimi E. In vitro dentin tubule occlusion and remineralization competence of various toothpastes. Arch Oral Biol. 2015;60(9):1246–53. 26092766 10.1016/j.archoralbio.2015.05.012

[31] Moshaverinia A, Ansari S, Moshaverinia M, Roohpour N, Darr JA, Rehman I. Effects of incorporation of hydroxyapatite and fluoroapatite nanobioceramics into conventional glass ionomer cements (GIC). Acta Biomater. 2008;4(2):432–40. 17921077 10.1016/j.actbio.2007.07.011

[32] Lucas ME, Arita K, Nishino M. Toughness, bonding and fluoride-release properties of hydroxyapatite-added glass ionomer cement. Biomater. 2003;24(21):3787–94. 10.1016/s0142-9612(03)00260-6 12818551

[33] Yap AU, Pek YS, Kumar RA, Cheang P, Khor KA. Experimental studies on a new bioactive material: HAIonomer cements. Biomater. 2002;23(3):955–62. 10.1016/s0142-9612(01)00208-3 11774854

[34] Lee JJ, Lee YK, Choi BJ, Lee JH, Choi HJ, Son HK, Hwang JW, Kim SO. Physical properties of resin-reinforced glass ionomer cement modified with micro and nano-hydroxyapatite. J Nanosci Nanotechnol. 2010;10(8):5270–6. 21125881 10.1166/jnn.2010.2422

[35] Wan Jusoh WN, Matori KA, Mohd Zaid MH, Zainuddin N, Ahmad Khiri MZ, Abdul Rahman NA, Abdul Jalil R, Kul E. Incorporation of hydroxyapatite into glass ionomer cement (GIC) formulated based on Alumino-Silicate-Fluoride glass ceramics from waste materials. 2021, 14(4):954. 10.3390/ma14040954 PMC7923024 33670465

[36] Ivanišević A, Rajić VB, Pilipović A, Par M, Ivanković H, Baraba A. Compressive strength of conventional glass ionomer cement modified with TiO2 Nano-Powder and Marine-Derived hap Micro-Powder. 2021, 14(17):4964. 10.3390/ma14174964 PMC8434552 34501056

[37] Zhu K, Zheng L, Xing J, Chen S, Chen R, Ren L. Mechanical, antibacterial, biocompatible and microleakage evaluation of glass ionomer cement modified by nanohydroxyapatite/polyhexamethylene Biguanide. Dent Mater J. 2022;41(2):197–208. 34759126 10.4012/dmj.2021-096

[38] Walsh WR, Guzelsu N. Compressive properties of cortical bone: mineral-organic interfacial bonding. Biomater. 1994;15(2):137–45. 10.1016/0142-9612(94)90263-1 8011860

[39] Moheet IA, Luddin N, Ab Rahman I, Masudi SM, Kannan TP, Abd Ghani NRNJP, Composites P. Novel nano-hydroxyapatite-silica–added glass ionomer cement for dental application: evaluation of surface roughness and sol-sorption. 2020, 28(5):299–308.

[40] Zaki ZM, Niazy MA, Zaazou MH, Nagi SM, Elkassas DW. Effect of incorporation of nano-hydroxyapatite particles on the clinical performance of conventional and resin-modified glass ionomer cement in class V cavities: split-mouth, randomized controlled trial. Bull Natl Res Cent. 2021;45(1):199.

[41] Kumari PD, Khijmatgar S, Chowdhury A, Lynch E, Chowdhury CR. Factors influencing fluoride release in atraumatic restorative treatment (ART) materials: A review. J Oral Biol Craniofac Res. 2019;9(4):315–20. 31334004 10.1016/j.jobcr.2019.06.015 PMC6624238

[42] Jingarwar MM, Pathak A, Bajwa NK, Sidhu HS. Quantitative assessment of fluoride release and recharge ability of different restorative materials in different media: an in vitro study. J Clin Diagn Res. 2014;8(12):Zc31–34. 25654027 10.7860/JCDR/2014/9985.5275 PMC4316333

[43] Murugan R, Yazid F, Nasruddin NS, Anuar NNM. Effects of nanohydroxyapatite incorporation into glass ionomer cement (GIC). 2022, 12(1):9.

[44] Pardi M, da Cunha BM, Cunha HM, Marques MES, Ribeiro KL, Cruz CE, Costa CR, Lepri CP, de Castro DT. Correlation between fluoride release, surface hardness and diametral tensile strength of restorative glass ionomer cements. J Clin Exp Dent. 2024;16(5):e610–5. 38988758 10.4317/jced.61499 PMC11231896

[45] Mohd Pu’ad NAS, Koshy P, Abdullah HZ, Idris MI, Lee TC. Syntheses of hydroxyapatite from natural sources. Heliyon. 2019;5(5):e01588. 31080905 10.1016/j.heliyon.2019.e01588 PMC6507053

[46] Hamdi K, Elsebaai A, Abdelshafi MA, Hamama HH. Remineralization and anti-demineralization effect of orthodontic adhesives on enamel surrounding orthodontic brackets: a systematic review of in vitro studies. BMC Oral Health. 2024;24(1):1446. 39609782 10.1186/s12903-024-05237-y PMC11603835

