# Supplementary material for: The influence of incorporation of hydroxyapatite/collagen nanocomposite into glass ionomer cement on surface roughness, microhardness, and fluoride-ion release potential
Source: BMC Oral Health. 2025 Nov 1;25:1721. doi: 10.1186/s12903-025-07080-1 (PMC12579407; doi:10.1186/s12903-025-07080-1)
Supplement: Supplementary file 2 — Supplementary Material 2. [file 12903_2025_7080_MOESM2_ESM.docx]

**Results**

table ():comparison of microhardness and roughness between studied groups

|  | | N | Mean | Std. Deviation | Std. Error | 95% Confidence Interval for Mean | | Minimum | Maximum |
| --- | --- | --- | --- | --- | --- | --- | --- | --- | --- |
|  |  |  |  |  |  | Lower Bound | Upper Bound |  |  |
| microhardness | Control | 15 | 42.5933 | 4.67951 | 1.20824 | 40.0019 | 45.1848 | 32.40 | 49.60 |
|  | 5 % (70:30 HA:Col) | 15 | 54.8400 | 10.88175 | 2.80966 | 48.8139 | 60.8661 | 38.20 | 78.30 |
|  | 10 % (70:30 HA:Col) | 15 | 58.9133 | 16.89019 | 4.36103 | 49.5599 | 68.2668 | 41.40 | 97.70 |
|  | 5 % (50:50 HA:Col) | 15 | 58.3013 | 8.80200 | 2.27267 | 53.4269 | 63.1757 | 46.80 | 76.60 |
|  | 10 % (50:50 HA:Col) | 15 | 55.9193 | 10.32108 | 2.66489 | 50.2037 | 61.6350 | 39.62 | 72.42 |
|  | Total | 75 | 54.1135 | 12.29724 | 1.41996 | 51.2841 | 56.9428 | 32.40 | 97.70 |
| roughness | Control | 15 | .5187 | .19350 | .04996 | .4115 | .6258 | .27 | .92 |
|  | 5 % (70:30 HA:Col) | 15 | .4293 | .10566 | .02728 | .3708 | .4878 | .20 | .64 |
|  | 10 % (70:30 HA:Col) | 15 | .5113 | .10636 | .02746 | .4524 | .5702 | .34 | .68 |
|  | 5 % (50:50 HA:Col) | 15 | .4733 | .11337 | .02927 | .4106 | .5361 | .25 | .72 |
|  | 10 % (50:50 HA:Col) | 15 | .7153 | .16999 | .04389 | .6212 | .8095 | .36 | .90 |
|  | Total | 75 | .5296 | .17029 | .01966 | .4904 | .5688 | .20 | .92 |

| **ANOVA** | | | | | | |
| --- | --- | --- | --- | --- | --- | --- |
|  | | Sum of Squares | df | Mean Square | F | Sig. |
| microhardness | Between Groups | 2656.191 | 4 | 664.048 | 5.447 | .001 |
|  | Within Groups | 8534.239 | 70 | 121.918 |  |  |
|  | Total | 11190.431 | 74 |  |  |  |
| roughness | Between Groups | .723 | 4 | .181 | 8.884 | .000 |
|  | Within Groups | 1.423 | 70 | .020 |  |  |
|  | Total | 2.146 | 74 |  |  |  |

| **Multiple Comparisons** | | | | | | | |
| --- | --- | --- | --- | --- | --- | --- | --- |
| Tukey HSD | | | | | | | |
| Dependent Variable | (I) group | (J) group | Mean Difference (I-J) | Std. Error | Sig. | 95% Confidence Interval | |
|  |  |  |  |  |  | Lower Bound | Upper Bound |
| microhradness | Control | 5 % (70:30 HA:Col) | -12.24667^*^ | 4.03184 | .027 | -23.5364 | -.9569 |
|  |  | 10 % (70:30 HA:Col) | -16.32000^*^ | 4.03184 | .001 | -27.6098 | -5.0302 |
|  |  | 5 % (50:50 HA:Col) | -15.70800^*^ | 4.03184 | .002 | -26.9978 | -4.4182 |
|  |  | 10 % (50:50 HA:Col) | -13.32600^*^ | 4.03184 | .013 | -24.6158 | -2.0362 |
|  | 5 % (70:30 HA:Col) | Control | 12.24667^*^ | 4.03184 | .027 | .9569 | 23.5364 |
|  |  | 10 % (70:30 HA:Col) | -4.07333 | 4.03184 | .850 | -15.3631 | 7.2164 |
|  |  | 5 % (50:50 HA:Col) | -3.46133 | 4.03184 | .911 | -14.7511 | 7.8284 |
|  |  | 10 % (50:50 HA:Col) | -1.07933 | 4.03184 | .999 | -12.3691 | 10.2104 |
|  | 10 % (70:30 HA:Col) | Control | 16.32000^*^ | 4.03184 | .001 | 5.0302 | 27.6098 |
|  |  | 5 % (70:30 HA:Col) | 4.07333 | 4.03184 | .850 | -7.2164 | 15.3631 |
|  |  | 5 % (50:50 HA:Col) | .61200 | 4.03184 | 1.000 | -10.6778 | 11.9018 |
|  |  | 10 % (50:50 HA:Col) | 2.99400 | 4.03184 | .946 | -8.2958 | 14.2838 |
|  | 5 % (50:50 HA:Col) | Control | 15.70800^*^ | 4.03184 | .002 | 4.4182 | 26.9978 |
|  |  | 5 % (70:30 HA:Col) | 3.46133 | 4.03184 | .911 | -7.8284 | 14.7511 |
|  |  | 10 % (70:30 HA:Col) | -.61200 | 4.03184 | 1.000 | -11.9018 | 10.6778 |
|  |  | 10 % (50:50 HA:Col) | 2.38200 | 4.03184 | .976 | -8.9078 | 13.6718 |
|  | 10 % (50:50 HA:Col) | Control | 13.32600^*^ | 4.03184 | .013 | 2.0362 | 24.6158 |
|  |  | 5 % (70:30 HA:Col) | 1.07933 | 4.03184 | .999 | -10.2104 | 12.3691 |
|  |  | 10 % (70:30 HA:Col) | -2.99400 | 4.03184 | .946 | -14.2838 | 8.2958 |
|  |  | 5 % (50:50 HA:Col) | -2.38200 | 4.03184 | .976 | -13.6718 | 8.9078 |
| roughness | Control | 5 % (70:30 HA:Col) | .08933 | .05207 | .431 | -.0565 | .2351 |
|  |  | 10 % (70:30 HA:Col) | .00733 | .05207 | 1.000 | -.1385 | .1531 |
|  |  | 5 % (50:50 HA:Col) | .04533 | .05207 | .907 | -.1005 | .1911 |
|  |  | 10 % (50:50 HA:Col) | -.19667^*^ | .05207 | .003 | -.3425 | -.0509 |
|  | 5 % (70:30 HA:Col) | Control | -.08933 | .05207 | .431 | -.2351 | .0565 |
|  |  | 10 % (70:30 HA:Col) | -.08200 | .05207 | .518 | -.2278 | .0638 |
|  |  | 5 % (50:50 HA:Col) | -.04400 | .05207 | .915 | -.1898 | .1018 |
|  |  | 10 % (50:50 HA:Col) | -.28600^*^ | .05207 | .000 | -.4318 | -.1402 |
|  | 10 % (70:30 HA:Col) | Control | -.00733 | .05207 | 1.000 | -.1531 | .1385 |
|  |  | 5 % (70:30 HA:Col) | .08200 | .05207 | .518 | -.0638 | .2278 |
|  |  | 5 % (50:50 HA:Col) | .03800 | .05207 | .949 | -.1078 | .1838 |
|  |  | 10 % (50:50 HA:Col) | -.20400^*^ | .05207 | .002 | -.3498 | -.0582 |
|  | 5 % (50:50 HA:Col) | Control | -.04533 | .05207 | .907 | -.1911 | .1005 |
|  |  | 5 % (70:30 HA:Col) | .04400 | .05207 | .915 | -.1018 | .1898 |
|  |  | 10 % (70:30 HA:Col) | -.03800 | .05207 | .949 | -.1838 | .1078 |
|  |  | 10 % (50:50 HA:Col) | -.24200^*^ | .05207 | .000 | -.3878 | -.0962 |
|  | 10 % (50:50 HA:Col) | Control | .19667^*^ | .05207 | .003 | .0509 | .3425 |
|  |  | 5 % (70:30 HA:Col) | .28600^*^ | .05207 | .000 | .1402 | .4318 |
|  |  | 10 % (70:30 HA:Col) | .20400^*^ | .05207 | .002 | .0582 | .3498 |
|  |  | 5 % (50:50 HA:Col) | .24200^*^ | .05207 | .000 | .0962 | .3878 |
| *. The mean difference is significant at the 0.05 level. | | | | | | | |

table ():

| **Estimates** | | | | |
| --- | --- | --- | --- | --- |
| Dependent Variable: flouride | | | | |
| time.assessment | Mean | Std. Error | 95% Confidence Interval | |
|  |  |  | Lower Bound | Upper Bound |
| 28 Days | 3.790 | .021 | 3.749 | 3.831 |
| 14 Days | 6.080 | .021 | 6.039 | 6.121 |
| 7 Days | 6.987 | .021 | 6.946 | 7.028 |
| 1 Day | 10.016 | .021 | 9.975 | 10.057 |

table (): mixed linear model for predictors of flouride

| **Tests of Between-Subjects Effects** | | | | | | |
| --- | --- | --- | --- | --- | --- | --- |
| Dependent Variable: flouride | | | | | | |
| Source | Type III Sum of Squares | df | Mean Square | F | Sig. | Partial Eta Squared  (effect size) |
| Corrected Model | 2166.020^a^ | 19 | 114.001 | 3500.411 | .0001* | .996 |
| Intercept | 13540.263 | 1 | 13540.263 | 415754.820 | .0001* | .999 |
| groups | 453.696 | 4 | 113.424 | 3482.691 | .0001* | .980 |
| Time of assessment | 1494.828 | 3 | 498.276 | 15299.599 | .0001* | .994 |
| groups * Time of assessment | 217.497 | 12 | 18.125 | 556.521 | .0001* | .960 |
| Error | 9.119 | 280 | .033 |  |  |  |
| Total | 15715.403 | 300 |  |  |  |  |
| Corrected Total | 2175.139 | 299 |  |  |  |  |
| a. R Squared = .996 (Adjusted R Squared = .996) | | | | | | |

| **Pairwise Comparisons** | | | | | | |
| --- | --- | --- | --- | --- | --- | --- |
| Dependent Variable: flouride | | | | | | |
| (I) time. assessment | (J) time assessment | Mean Difference (I-J) | Std. Error | Sig.^b^ | 95% Confidence Interval for Difference^b^ | |
|  |  |  |  |  | Lower Bound | Upper Bound |
| 28 Days | 14 Days | -2.290^*^ | .029 | .000 | -2.369 | -2.212 |
|  | 7 Days | -3.198^*^ | .029 | .000 | -3.276 | -3.119 |
|  | 1 Days | -6.226^*^ | .029 | .000 | -6.305 | -6.148 |
| 14 Days | 28 Days | 2.290^*^ | .029 | .000 | 2.212 | 2.369 |
|  | 7 Days | -.907^*^ | .029 | .000 | -.986 | -.829 |
|  | 1 Days | -3.936^*^ | .029 | .000 | -4.014 | -3.858 |
| 7 Days | 28 Days | 3.198^*^ | .029 | .000 | 3.119 | 3.276 |
|  | 14 Days | .907^*^ | .029 | .000 | .829 | .986 |
|  | 1 Days | -3.029^*^ | .029 | .000 | -3.107 | -2.950 |
| 1 Day | 28 Days | 6.226^*^ | .029 | .000 | 6.148 | 6.305 |
|  | 14 Days | 3.936^*^ | .029 | .000 | 3.858 | 4.014 |
|  | 7 Days | 3.029^*^ | .029 | .000 | 2.950 | 3.107 |
| Based on estimated marginal means | | | | | | |
| *. The mean difference is significant at the .05 level. | | | | | | |
| b. Adjustment for multiple comparisons: Bonferroni. | | | | | | |

| **Univariate Tests** | | | | | | |
| --- | --- | --- | --- | --- | --- | --- |
| Dependent Variable: flouride | | | | | | |
|  | Sum of Squares | df | Mean Square | F | Sig. | Partial Eta Squared |
| Contrast | 1494.828 | 3 | 498.276 | 15299.599 | .000 | .994 |
| Error | 9.119 | 280 | .033 |  |  |  |
| The F tests the effect of time.assessment. This test is based on the linearly independent pairwise comparisons among the estimated marginal means. | | | | | | |

| **2. groups * time assessment** | | | | | |
| --- | --- | --- | --- | --- | --- |
| Dependent Variable: fluoride | | | | | |
| groups | Time assessment | Mean | Std. Error | 95% Confidence Interval | |
|  |  |  |  | Lower Bound | Upper Bound |
| Control | 28 Days | 3.111 | .047 | 3.020 | 3.203 |
|  | 14 Days | 4.203 | .047 | 4.111 | 4.294 |
|  | 7 Days | 4.859 | .047 | 4.768 | 4.951 |
|  | 1 Day | 5.093 | .047 | 5.002 | 5.185 |
| 5 % (70:30 HA:Col) | 28 Days | 4.564 | .047 | 4.472 | 4.656 |
|  | 14 Days | 6.452 | .047 | 6.360 | 6.544 |
|  | 7 Days | 7.551 | .047 | 7.460 | 7.643 |
|  | 1 Day | 12.618 | .047 | 12.526 | 12.710 |
| 10 % (70:30 HA:Col) | 28 Days | 4.081 | .047 | 3.989 | 4.172 |
|  | 14 Days | 6.210 | .047 | 6.118 | 6.302 |
|  | 7 Days | 7.675 | .047 | 7.584 | 7.767 |
|  | 1 Day | 11.098 | .047 | 11.006 | 11.190 |
| 5 % (50:50 HA:Col) | 28 Days | 3.571 | .047 | 3.479 | 3.662 |
|  | 14 Days | 6.422 | .047 | 6.330 | 6.514 |
|  | 7 Days | 7.380 | .047 | 7.288 | 7.472 |
|  | 1 Day | 10.553 | .047 | 10.462 | 10.645 |
| 10 % (50:50 HA:Col) | 28 Days | 3.621 | .047 | 3.530 | 3.713 |
|  | 14 Days | 7.113 | .047 | 7.022 | 7.205 |
|  | 7 Days | 7.471 | .047 | 7.379 | 7.562 |
|  | 1 Day | 10.717 | .047 | 10.625 | 10.808 |
